# Compression pre-stapler firing and post-ignition wait during sleeve gastrectomy: a prospective randomized trial

**DOI:** 10.1590/1516-3180.2023.0163.140823

**Published:** 2023-12-15

**Authors:** Medeni Sermet

**Affiliations:** I MD, PhD. Department of General Surgery, Goztepe Prof. Dr. Suleyman Yalcin City Hospital, Medeniyet University, Istanbul, Turkey.

**Keywords:** Gastrectomy, Stapling, Hemorrhage, Surgical stapling, Bariatrics, Surgical staple, Morbid obesities, Bariatric surgery

## Abstract

**BACKGROUND:**

Insufficient research exists on the stapling technique in and duration of laparoscopic sleeve gastrectomy (LSG).

**OBJECTIVES:**

This study aimed to assess the clinical outcomes using a 30-second precompression and post-firing waiting time without extra support for the stapling line.

**DESIGN AND SETTINGS:**

Randomized controlled prospective study at a university hospital.

**METHODS:**

This study included 120 patients treated between January 2022 and February 2023. The patients were divided into the non-waiting group (T0) and waiting group (T1), each with 60 patients. Perioperative complications were analyzed using statistical tests.

**RESULTS:**

The waiting group (T1) showed a significant reduction in the number of intraoperative bleeding points requiring intervention compared with the non-waiting group (T0) (81 versus 134, P < 0.05). In T0, postoperative C-reactive protein (CRP) levels increased (P < 0.05) and hemoglobin levels decreased significantly (P <0.05). The study recorded 22 postoperative complications, accounting for 18.3% of all cases during the 30-day postoperative period.

**CONCLUSIONS:**

The study concluded that the 30 sec + 30 sec stapling technique reduces perioperative bleeding, length of stay, and serious complication rates and is practical and effective for LSG.

**CLINICAL TRIAL REGISTRATION:**

ClinicalTrials.gov with registration code NCT05703035; link: https://clinicaltrials.gov/ct2/show/NCT05703035.

## INTRODUCTION

Laparoscopic sleeve gastrectomy (LSG) is the preferred surgical option to address obesity and is the most widely used procedure.^1^ LSG offers several advantages including ease of learning compared to other procedures, short operation time, and minimal changes to the natural anatomy of the gastrointestinal system. Additionally, the surgical outcomes had positive effects on weight loss and comorbidities. However, despite technological advancements, the complication rate for leakage and bleeding remained between 0.5% and 2%.^2^ In 90% of cases, leaks occur at the sense angle, and they are likely related to technical errors during stapler firing.^3^ Techniques that strengthen the staple line to reduce complications place an economic burden on payment systems by increasing patient costs. Staple malformation is the main cause of leakage and bleeding.^4,5^

## OBJECTIVE

This study aimed to examine the potential effectiveness of precompression of 30 s before stapler firing and a waiting period of 30 s after firing, without utilizing any additional support or reinforcement for the staple line, in minimizing both intraoperative and postoperative complications. We hypothesized that the waiting period would result in optimal B formation, thereby reducing bleeding and leakage. Identifying factors such as staple size during LSG and firing technique can assist in improving patient care and optimizing bariatric center outcomes by predicting complications.

## METHODS

### Study design

A double-blind (patient, postoperative data collector, and statistician), randomized controlled prospective study on class III morbidly obese patients matched for body mass index (BMI) and comorbidities was conducted in a tertiary education and research hospital between January 2022 and February 2023. The study was approved by the ethics committee of the University of Medeniyet (decision no. 2021/0530, dated August 12, 2021), and the trial was registered at ClinicalTrials.gov with registration code NCT05703035.

Patients were randomly classified into two groups: T0 (patients who did not wait) and T1 (patients who waited). The patients underwent preoperative, intraoperative, and postoperative interventions based on the principles of multimodal enhanced recovery bariatric surgery (ERABS).

The patients underwent preoperative, intraoperative, and postoperative interventions according to the principles of multimodal enhanced recovery bariatric surgery (ERABS). Antithrombotic prophylaxis with enoxaparin was administered until postoperative day 14, and all the patients were followed up based on our routine enhanced recovery protocol, including oral intake beginning on postoperative day 1 and discharge planned on postoperative day 2.

Discharge Criteria:

■ Anamnesis
  - Visual analogue score < 4
  - No complaints of nausea or vomiting
  - Oral fluid intake > 1,500 ml in 24 hours
  - Moving and walking independently without support
  - No complaints of leg pain
■ Physical Examination
  - Abdominal examination is normal
  - Body fever < 38°C
  - Pulse rate < 100 bpm
  - Oxygen saturation (SatO2) > %95
  - Respiration rate: 10–16
  - Drainage < 50 ml
■ Laboratory Results
  - Postoperative hemoglobin decline < 2.0 g/dL
  - White blood cell (WBC) < 12 × 10^3/uL
  - C-reactive protein (CRP) < 20 mg/dL

Postoperative follow-up data were recorded by nursing staff and physicians’ assistants who were blinded to the procedures. Our prospective database included the documentation of all medical and surgical complications. In this study, intraoperative parameters, such as leakage, bleeding, reoperation and mortality rates, operative time, number of stapler shots, intraoperative bleeding, number of bleeding points treated with clips on the stapler line, and amount of blood in the aspirator and gauze, were recorded. Laboratory tests were requested from the patients on postoperative days 1, 7, and 30. Bleeding was defined as hemoglobin > 2 g/dL, pure blood drainage > 100 ml, or serohemorrhagic drainage > 200 ml and standing blood pressure < 20 mmHg. Parameters for leaks included purulent drainage from the drain, fever, tachycardia, increased respiratory rate, and severe epigastric pain.

### Study Population

With a Cohen's d effect size of 0.5, 46 participants were required in each group for a prospective randomized controlled study of sleeve gastrectomy using staple firing with and without precompression, with 80% power and a 5% alpha level. Assuming a potential 10% loss to follow-up, the required sample size was 102. A 12-month enrollment period was anticipated for patient recruitment. The sample size was increased to reach a total of 120 patients in both groups. The study included 120 patients (60 each in T0 and T1).

Inclusion criteria:

- Age: 18–65 years
- BMI > 40.0–49.90 kg/cm2
- Not using anticoagulant drugs
- Never underwent bariatric surgery before

Exclusion criteria:

- Patients who applied for revision surgery
- Patients with a history of thromboembolism
- Patients with known clotting disorders

The selected patients were given ample time to review the details of the study and answer questions. Those who agreed to participate voluntarily signed an informed consent form. Patients who declined to participate or were not eligible for the study were provided standard patient care according to the protocol.

### Interventions of the Study

#### Surgical Procedure and Stapler Technique

Each patient was administered 40 mg of enoxaparin subcutaneously 12 h before surgery. Pneumoperitoneum was created after routine placement of four ports. A Nathanson liver retractor was routinely used. Stomach dissection was performed using an energy device (LigaSure Atlas; Covidien LLC, United States).^6^ Gastric calibration was performed using a 38-French gastric bougie placed in the stomach. Gastric transection was initiated with continuous linear staples approximately 3 cm from the pylorus. In all patients, the first stapler was 60 mm black (leg length (4-4.5-5 mm), followed by 60 mm pink stapler (leg length 3-3.5-4 mm) (Endo GIA™ Articulating Reloads with Tri-staple™ Technology, Covidien LLC, United States of America). The last stapler was used, leaving a sufficient distance (approximately 1 cm) from the sense angle. After transection, the resected stomach was removed through a 15 mm trocar site. The gastric tube was pulled up to 37 cm, and a leak test was performed. This was performed using 120 mL of saline stained with methylene blue. No reinforcement support was used for the stapler line in any patient. A silicone drain was placed in the operative area for all patients.

In the waiting group, after the staple was locked into the stomach, compression was applied for 30 s, and firing was performed in four continuous motions (15 mm per movement). After firing was completed, the punch jaws were left compressed for another 30 s without opening, after which the jaws were opened and the process was completed. The first stapler was fired at 0°, the second at 9°, and a routine angulation of 18° was given to the third and subsequent staplers. In the non-waiting group, firing and cutting were performed without waiting after tissue locking with the stapler, without changing the order of use.

### Randomization

After the eligibility screening was conducted by the research coordinator, each patient was assigned a unique number using the hospital system. The randomization program (https://www.randomizer.org/) stratified patients into blocks 4 and 6, and all the randomized patients received care during the study period according to the intervention they were assigned. The study statistician, service follow-up doctor, care team, and patients were blinded to the procedure.

### Study Outcomes

The primary outcome of the study was whether waiting for the stapling procedure reduced the rates of bleeding and leakage during and after surgery. The secondary outcomes were the need for additional interventions outside of standard care, morbidity, mortality, and length of hospital stay without any reinforcement of the stapler line. Patients were followed up in the ward and as outpatients for up to 30 days postoperatively to determine whether they experienced any of the complications included in the composite outcome.

### Statistical analysis

Follow-up data were collected by a physician and a statistician who were blinded to the treatment groups. Mean and standard deviation was used to express continuous variables. The baseline characteristics of the patients in both groups were reported using descriptive statistics, such as frequency distributions, central tendency, and measures of distribution. Student's t-test was used for normally distributed numerical variables, the chi-square test was used for categorical variables, and the Mann–Whitney U test was used for non-parametric variables. The adjusted odds ratios (ORs) with 95% confidence intervals were presented as the results of the multivariate logistic regression analysis. Statistical significance was set at P < 0.05. Statistical analyses were performed using JMP 11 software (SAS Institute Inc., Cary, NC, USA).

## RESULTS

Both groups had similar demographic and clinical characteristics (Table 1).

**Table 1:** Statistical analysis of patients’ demographic and clinical characteristic features

Parameters		T0 (n = 60)	T1 (n = 60)	P
Gender (Female/Male)		48/12 (80.0%/20.0%)	50/10 (83.3%/16.7%)	0.498**
Age (years)		33.8 (20-59)	34.3(21-56)	0.439*
Height (cm)		159 (148–179)	158 (157–182)	0.632***
Weight (kg)		117.2 (105-165)	116.4(107-159)	0.454***
BMI (kg/cm^2^)		42.3 (40.1–49.2)	43.1(40.5, 2–48.9)	0.543***
Obesity-related comorbidity				
	T2D	22 (36.7%)	20 (32.7%)	0.434***
	Hypertension	11 (30.0%)	10 (16.3%)	0.657***
	OSAS	5 (8.3%)	6 (9.8%)	0.322***
	Hyperlipidemia	14 (23.3%)	15 (24.5%)	0.645***

BMI = body mass index; OSAS = obstructive sleep apnea syndrome; T2D = type 2 diabetes; categorical variables are expressed as n (%) and continuous variables as median (IQR); T0 = non-waiting group; T1 = waiting group.

* Student's t-test (mean, standard deviation);

** Chi-square test;

*** Mann–Whitney test; P < 0.05, considered statistically significant;.

**Figure 1:**
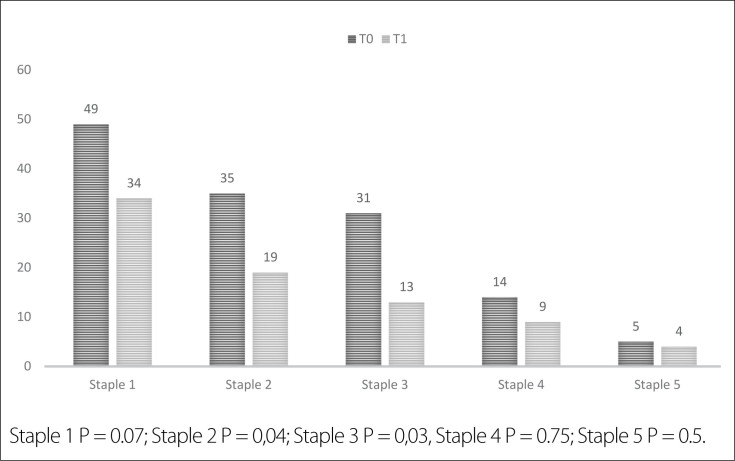
Plot of bleeding points on the punch line.

Patients with organ damage or bleeding unrelated to the stapling procedure performed during surgery were excluded. The number of bleeding points on the stapling line was assessed by reducing the intra-abdominal pressure to 8 mm Hg for 5 min. The waiting group (T1) showed significantly fewer stapling line bleeding points requiring intervention than the other group (81 versus 134, P < 0.05), resulting in a 28% better performance without additional measures. Metallic clips were used for hemostasis in all cases, and bleeding points were observed as staple firings in both groups (Figure 1). However, T1 had significantly fewer bleeding points at the second and third staple-firing stages (P < 0.05). Intraoperative blood loss was measured using an aspirator, and pressure was applied with gauze in some cases. T0 had a significantly greater intraoperative loss (P < 0.05); however, the overall loss was not significant. Further, T0 had a significantly shorter mean operation time of 8 min (P < 0.05).

As regards postoperative outcomes, patients with a decrease in hemoglobin level > 2 mg/dL after surgery in T0 had a higher incidence of bleeding than those in T1 (20% versus 8.6%). Two patients in T0 required 4 units of erythrocyte suspension transfusion (P < 0.05). Complications according to the Clavien–Dindo classification occurred in 22 cases (18.3% of all cases) within the 30-day postoperative period; however, no deaths were recorded. Additional interventions were performed in one patient in T0 because of ineffective drainage and in one patient in T1 because of fever caused by atelectasis. No leakage or thromboembolic events occurred during the 30-day follow-up in either group. Hospitalization duration was significantly longer in T0 than in T1 (P < 0.05) (Table 2).

**Table 2:** Analysis of patients’ intraoperative and postoperative data

Parameters		T0 (n = 60)	T1 (n = 60)	P
İntraoperative				
Operation time(minutes)		52.4 (7.8)*	64.1 (5.3)	< 0,001**
Number of staples used		5.12 (4-6)	5.26 (4-6)	0,452**
Number of bleeding points		134 (0-5)	81 (0-3)*	0,003**
Intraoperative blood loss (mL)		30 (15–25)	15 (10–25)*	0,001*
Clip for hemostasis (median)		3 (0–5)	2 (0–4)	0,116**
Number of patients without bleeding %		11	17	0,002**
Postoperative				
Drain Mean blood loss (ml)		119.4 (30-400)	114.6 (30-225)	0,301*
Clavien-Dindo Classification				
	Transfusion %	2 (3.3)	0*	0,042*
	Post Op bleeding %	12 (20)	5 (8.3)*	< 0,001*
	Postop leak	0	0	1,00*
	Thromboembolic event	0	0	1,00*
	Hematoma %	1(1.7)	0	0,754*
	Vomiting %	1 (1.7)	2 (3.3)	0,342*
	Fever %	0	1	0,754*
Length of stay (days)		2.35 (2-5)	2.14 (2-3)*	0,02*
Gastric tissue thickness (mm)		0.27 (0.3)	0.27 (0.2)	0,978*

Categorical variables are expressed as n (%) and continuous variables as median (IQR);

* Student's t-test (mean, standard deviation);

** Mann–Whitney test;

P < 0.05, considered statistically significant; T0 = non-waiting group; T1 = waiting group.

Regarding laboratory values, the mean hemoglobin decrease was greater in T0 than in T1 (1.9 g/dL vs. 1.5 g/dL, P < 0.05). The acute-phase reactant CRP levels were significantly higher in T0 (P < 0.05). The WBC count and coagulation values increased in both groups after surgery; however, the difference was not statistically significant. Ultrasound controls at 1 week and 1 month post-surgery were normal. Average gastric wall thickness, as determined by pathological evaluation, did not significantly correlate with complications (Table 3).

**Table 3:** Univariate analysis of laboratory and imaging tests of patients

Parameters		T0 (n = 60)	T1 (n = 60)	P
Preoperative				
	WBC (10^3 /uL)	7.7 (2.2)	7.2 (3.5)	0,345*
	Hemoglobin (g/dL)	13.2 (3.2)	13.4 (3.1)	0,467*
	PLT (10^3 /uL)	231 (114)	234 (98)	0,629**
	CRP (mg/L)	2.0 (1.8)	2.1 (0.7)	0.784*
	INR	0,98 (0,1)	0.98 (0,1)	0,493**
	PT (sn)	14.0 (0.2)	14.1 (0.3)	0,618**
	PTT (sn)	83.2 (8.2)	82.9 (7.0)	0,382**
	Fibrinogen (mg/dL)	270 (78)	274 (72)	0,234**
	USG	N	N	1.00**
Postoperative				
	WBC (10^3 /uL)	13.7 (4.9)	12.9 (6.2)	0,237*
	Hemoglobin (g/dL)	11.2 (0.6)*	11.9 (1.1)	0,025*
	PLT (10^3 /uL)	244 (102)	239 (98)	0,532**
	CRP (mg/L)	28.46 (8.5-110.6)	21.3 (7.3-87.6)*	0.014*
	INR	0.99 (0.05)	1.0 (0.03)	0.493**
	PT (sn)	14.2 (0.2)	14.4 (0.3)	0.578**
	PTT (sn)	84.2 (7.0)	83.9 (7.3)	0.382**
	Fibrinogen (mg/dL)	274 (72)	277 (74)	0.234**
	USG	1 (hematoma)	N	1.00*

WBC = white blood cell; PLT = platelet count; CRP = C-reactive protein; INR = international normalized ratio; PT = prothrombin time; PTT = Partial thromboplastin time; USG = ultrasonography; Categorical variables were expressed as n (%) and continuous variables as median (IQR);

* Student's t-test (mean, standard deviation);

** Mann–Whitney test;

P < 0.05 was considered statistical significance; T0 = non-waiting group; T1 = waiting group.

## DISCUSSION

Surgical staplers are commonly used in various surgical procedures to facilitate rapid and effortless tissue division and closure. Its use in bariatric surgery is considered the gold standard. Studies have shown that the use of reinforcing products on the stapling line is beneficial.^7^ Stapler manufacturers suggest that tissue can be clamped between the jaws of the stapler and cut in a flat position. However, there are no recommendations regarding waiting time.^8^

Research showing the beneficial results of waiting for a certain amount of time before stapling is limited.^9^ The optimal waiting and stapling times are unclear. Based on experience, some surgeons recommended waiting a while before firing the stapler to ensure adequate tissue compression for hemostasis.^10^ During LSG, bleeding may occur along the stapler line, which may require additional measures such as suturing the edges of the stapler line, using clips, or using electrocautery to stop the bleeding. Difficulty in diagnosis and indecisiveness in timely intervention during the postoperative period can affect morbidity and hospital stay.^11^ An animal model study has shown that the number of bleeding points from the stapler line can be significantly reduced by using waiting times of 0, 1, and 5 minutes before firing as a stapling technique.^12^

In our study, staple line bleeding was observed in 17 (14.1%) patients, with 12 patients in T0 and 5 patients in T1, respectively. These results indicate that the current rate is higher than that previously reported in the literature. We believe that this is due to our comprehensive assessment, which included variables that we believe were associated with bleeding and broad in scope. Two patients in the non-waiting group underwent transfusion because of bleeding, and the other patients were managed conservatively. Better bleeding outcomes were achieved in T1. This can be attributed to the compression–wait–firing–wait–separation technique used, which compresses the tissue to obtain a flatter and thinner tissue, reduces staple slippage from the tissue during firing, and promotes optimal staple formation.

Intraluminal bleeding cannot be observed intraoperatively prior to endoscopic inspection after staple firing. Bleeding at the staple line may indicate bleeding within the lumen of an organ or structure.^12,13^ Delaying the firing of a staple for a period of time is a simple method to reduce staple line bleeding, which may be associated with a decrease in the likelihood of intraluminal bleeding.^14^ In our study, after a total of 1 min of waiting and approximately 10 min of postoperative observation, no evidence of localized or diffuse ischemia was observed in the gastric tissue. This may be because of the thicker stomach tissue and abundant blood supply. Choosing an appropriate wait time for ignition further helps prevent tissue tension and bending during the procedure. In a study of distal pancreatectomy, this time was approximately 5 minutes.^15^

Major postoperative morbidity after LSG is often associated with staple line leakage, which has two main causes: ischemic or mechanical and technical aspects related to incorrect firing of the stapler and the type of cartridge used.^16^ Generally, the leakage rate after LSG is 1–2.7%; however, in our study, no leaks were observed in either group, probably owing to the sample size.

During the postoperative follow-up, 22 patients (18.6% of the total patients) had complications within the first 30 days after surgery. The type and frequency of these complications were similar to those reported in previous research studies.^17,18^

In a limited number of studies on distal pancreatectomy, a waiting time of 10 min has been shown to reduce tissue slippage as the staple legs penetrate the tissue, resulting in proper tissue compression and a smooth staple line by allowing fluid drainage.^19^ However, we did not find similar studies on gastric or intestinal tissue in the literature. In studies related to gastric tissue thickness, research has shown that tissue thickness decreases from the antrum to the proximal area, which is crucial in staple selection.^20^ We used Tri-Staple technology in all of our patients. Owing to the thicker antral tissue, we chose the first cartridge to be black and all subsequent cartridges to be purple. The average thickness of the stomach wall in our study was measured to be 2.7 mm. Our results suggest that appropriate staple selection in combination with waiting time may reduce bleeding and complication development.

The effects of tissue precompression have been determined in limited studies related to colorectal and pancreatic surgeries.^21,22^ However, the optimal waiting time remains unclear. In colorectal surgery, only data on precompression are available. The difference in our application was that we waited both during precompression and compression after firing. Therefore, we believe that the staples formed an optimal B-formation after firing and that the pressure on the tissue prevented protrusion between the staple teeth. Minimal disruption of tissue integrity was associated with reduced bleeding and leakage.

Overall, these findings highlight that stapling techniques should be considered in bariatric surgery to minimize postoperative bleeding and improve patient outcomes.

This study had some limitations. First, although stapler malformation is believed to be the primary cause of bleeding and leakage, whether optimal stapler formation is associated with improved clinical outcomes remains unclear. Second, the potential effects of precompression on the gastric wall, such as vascularization, bleeding, and tissue damage, were not evaluated. However, these factors are critical and require further investigation. Last, the lack of studies with longer dwell times limits the ability to compare and determine the most effective dwell time.

## CONCLUSION

Our study suggests that a 30-second precompression, along with a 30-second post-fire waiting period, possibly results in improved staple formation. In addition, precompression time is a critical factor in optimizing staple formation. Further, the removal of the device from the tissue after the waiting period is shown to have a significant effect on bleeding, hospital stay, and recovery.
